# Supplementary material for: MetaQTL: a package of new computational methods for the meta-analysis of QTL mapping experiments
Source: BMC Bioinformatics. 2007 Feb 8;8:49. doi: 10.1186/1471-2105-8-49 (PMC1808479; doi:10.1186/1471-2105-8-49)
Supplement: Additional File 7 — MetaQTL Package : jar file and tutorial. This Zip archive contains both the MetaQTL JAR file and the files of the tutorial. [file 1471-2105-8-49-S7.zip › org.inra.metaqtl/doc/org/metaqtl/graph/ChromShape.html]

ChromShape


|  |  |  |
| --- | --- | --- |
| **Overview** \| **Package** \| **Class** \| **Use** \| **Tree** \| **Deprecated** \| **Index** \| **Help** | |  |
| **PREV CLASS**   **NEXT CLASS** | **FRAMES**    **NO FRAMES**     **All Classes** |  |
| SUMMARY: NESTED \| FIELD \| CONSTR \| METHOD | DETAIL: FIELD \| CONSTR \| METHOD |  |


---


## org.metaqtl.graph Class ChromShape

```
java.lang.Object
  org.metaqtl.graph.GraphShape
      org.metaqtl.graph.ChromShape
```

---

``` public class ChromShape extends GraphShape ```

---

| **Field Summary** | |
| --- | --- |
| `static int` | `LEFT_LABEL` |
| `static int` | `RIGHT_LABEL` |

**Fields inherited from class org.metaqtl.graph.GraphShape**

`height, width, x, y`


**Constructor Summary**

`ChromShape()`


| **Method Summary** | |
| --- | --- |
| `void` | `buildShape(java.awt.Graphics2D graph, java.lang.Object object)` |
| `void` | `draw(java.awt.Graphics2D graph)`             Draws the shape. |
| `ChromAxe` | `getChromAxe()` |
| `double` | `getChromWidth()` |
| `int` | `getLabelSide()` |
| `java.awt.geom.Point2D` | `getMrkTickPoint2D(int mrkIdx, boolean reverse)` |
| `double` | `getXChrom()` |
| `double` | `getYMax()` |
| `double` | `getYMin()` |
| `void` | `setLabelSide(int side)` |

**Methods inherited from class org.metaqtl.graph.GraphShape**

`getHeight, getWidth`

**Methods inherited from class java.lang.Object**

`clone, equals, finalize, getClass, hashCode, notify, notifyAll, toString, wait, wait, wait`

**Field Detail**

### RIGHT\_LABEL

```
public static final int RIGHT_LABEL
```

**See Also:**: Constant Field Values

---


### LEFT\_LABEL

```
public static final int LEFT_LABEL
```

**See Also:**: Constant Field Values


**Constructor Detail**

### ChromShape

```
public ChromShape()
```


**Method Detail**

### buildShape

```
public void buildShape(java.awt.Graphics2D graph,
                       java.lang.Object object)
```

:   **Specified by:**: `buildShape` in class `GraphShape`
:   **Parameters:**: `chrom` -

---


### draw

```
public void draw(java.awt.Graphics2D graph)
```

:   **Description copied from class: `GraphShape`**
:   Draws the shape.

    :   **Specified by:**: `draw` in class `GraphShape`

---


### getXChrom

```
public double getXChrom()
```

:   **Returns:**

---


### setLabelSide

```
public void setLabelSide(int side)
```

---


### getChromWidth

```
public double getChromWidth()
```

:   **Returns:**

---


### getChromAxe

```
public ChromAxe getChromAxe()
```

:   **Returns:**

---


### getYMin

```
public double getYMin()
```

:   **Specified by:**: `getYMin` in class `GraphShape`

---


### getYMax

```
public double getYMax()
```

:   **Specified by:**: `getYMax` in class `GraphShape`

---


### getMrkTickPoint2D

```
public java.awt.geom.Point2D getMrkTickPoint2D(int mrkIdx,
                                               boolean reverse)
```

:   **Parameters:**: `mrkIdx` - **Returns:**

---


### getLabelSide

```
public int getLabelSide()
```

:   **Returns:**


---


|  |  |  |
| --- | --- | --- |
| **Overview** \| **Package** \| **Class** \| **Use** \| **Tree** \| **Deprecated** \| **Index** \| **Help** | |  |
| **PREV CLASS**   **NEXT CLASS** | **FRAMES**    **NO FRAMES**     **All Classes** |  |
| SUMMARY: NESTED \| FIELD \| CONSTR \| METHOD | DETAIL: FIELD \| CONSTR \| METHOD |  |


---
